# Economic analysis of producing vital statistics using civil registration data in Lao People’s Democratic Republic

**DOI:** 10.1186/s41043-019-0184-2

**Published:** 2019-10-18

**Authors:** Samuel Mills, Daniel Amponsah

**Affiliations:** World Bank Group, 1818 H Street, NW, Washington, DC, 20433 USA

## Abstract

The government of Lao People’s Democratic Republic (PDR) is currently in the preparation stage of a 5-year project that will establish an electronic civil registration and vital statistics (CRVS) system. The authors of this paper adapted a framework for economic analysis developed by Jimenez-Soto et al. (Jimenez-Soto et al., PLoS ONE 9(8): e106234, 2014) to assess the cost-effectiveness of producing vital statistics in Lao PDR using data from a complete electronic CRVS system, compared to using data from other sources, such as the 2015 Population and Housing Census and the 2017 Lao Social Indicator Survey (LSIS). Of 20 types of vital statistics (including birth statistics, fertility rates, and death statistics), a complete and accurate CRVS system can produce all 20 of these vital statistics, while the 2015 Census can produce 17, and the 2017 LSIS and the current civil registration system can produce 4 each. A cost-effectiveness analysis of different data sources for producing vital statistics over a 20-year projection showed that a complete and accurate CRVS system ranked best, followed by population census and population-based survey. In addition to enabling vital statistics to be produced cost-effectively, a robust civil registration system would also support improving the efficiency of public service delivery, leading to further cost savings for the country.

## Main text

### Background

As data are mined to inform evidence-based decision-making in both the private and public sectors, one data source with untapped development potential in low- and middle-income countries has been civil registration and vital statistics (CRVS). Data collected through civil registration -- *the universal, continuous, permanent, and compulsory recording of vital events occurring in a country’s population according to the legal requirements of each country* [1]-- are used to compile vital statistics defined as *the collection of statistics on vital events in a lifetime of a person as well as relevant characteristics of the events themselves and of the person and persons concerned* [2]. Besides civil registration, other data sources for generating vital statistics are population census, population-based survey, demographic surveillance system, and sample registration system. Nonetheless, civil registration, when it is complete and accurate, is considered the most valuable and least expensive data source for vital statistics [3].

Efforts are being made to improve CRVS in low- and middle-income countries, which has legal and administrative benefits beyond supporting the production of vital statistics [4–7]. Nevertheless, as Jimenez-Soto et al. [8] pointed out, a systematic framework for assessing the costs and benefits of the various data sources for producing vital statistics had been lacking. Accordingly, they proposed a systematic framework for such an assessment to inform investment decisions on the instruments for collecting data. To our knowledge, the framework has not been tested in a real country.

### Rationale and objective

The Global CRVS Scaling up Investment Plan 2015–2024 published by the World Bank in 2014 estimated that an average of US$1.5 per capita (which could be higher for countries with rudimentary CRVS systems) is required to strengthen CRVS systems in 73 low- and middle-income countries. However, the cost for producing vital statistics was not estimated [9]. Sustainable Development Solutions Network, a UN global initiative to mobilize scientific and technological expertise to promote practical development solutions, estimated that it would cost US$1 billion annually for 77 International Development Association [10] countries to improve national statistics systems to adequately monitor the Sustainable Development Goals but did not provide a breakdown of the cost specifically for vital statistics due to the paucity of detailed national costing data [11]. Further, in March 2019, the University of Melbourne published a CRVS Costing Tool which can be employed to estimate the cost for strengthening CRVS systems [12].

The Government of Lao People’s Democratic Republic (PDR) requested financing from the World Bank to establish a CRVS system. As part of the project appraisal, economic and financial analyses were required to be carried out to inform the government’s decision to invest in improving the CRVS system. The project’s interventions are expected to generate direct, indirect, and above all, intangible benefits to target beneficiaries (children, women, men) by establishing a functional electronic Civil Management Information System and thereby improving coverage of civil registration of life events, including births, deaths, marriages, divorces, and migration, in Lao PDR. Improving a CRVS system has three main advantages: (i) it provides legal status and documentation for accessing social services such as education, health, social welfare, and financial services; (ii) it helps to improve governance and administrative functions such as helping to identify the poor to provide them with target services and serves as a critical source of information for the effective and efficient delivery of public services including civil service, social registry, pensions, social security, passport, transportation or driver’s license, taxes, health care, finance, education, voter rolls, immigration; and (iii) it provides data for generating vital statistics for the monitoring of national development plans at national and subnational level. A cost-benefit analysis (i.e., determining whether dollar benefits of the Project is likely to outweigh dollar costs) could not be carried out since it is difficult to monetize the direct, indirect, and intangible benefits of civil registration for the legal and administrative advantages noted above. However, analyzing the cost-effectiveness of producing vital statistics from civil registration data compared to other data sources is possible.

This paper seeks to apply Jimenez-Soto et al.’s [8] proposed framework to assess the cost-effectiveness of producing vital statistics in Lao PDR using data from a complete electronic CRVS system, compared to using data from other sources, such as the 2015 Population and Housing Census and the 2017 Lao Social Indicator Survey (LSIS). The lower the cost per quality-adjusted capita of a data collection instrument, the more cost-effective the instrument.

### Methods

The authors of this paper adapted Jimenez-Soto et al.’s framework, using real data from Lao PDR to perform a cost-effectiveness analysis (CEA) of the sources of data for generating vital statistics in the country. The four elements of the framework are (1) identifying the alternative sources of data to be evaluated, (2) identifying and measuring the outcomes of the alternatives to be evaluated, (3) identifying and measuring the costs of the alternatives to be evaluated, and (4) conducting an economic assessment of the alternatives to be evaluated.

#### Identifying the alternative sources of data to be evaluated

The current sources of data for producing vital statistics in Lao PDR are population census and population-based survey. These two data sources are selected as alternatives to be evaluated since Lao PDR has recently conducted the last two in 2015 and 2017, respectively. Moreover, the current coverage of civil registration is low. For instance, birth registration is only paper-based, with no electronic system in place. As such, in the calendar year 2017 for example, 61,416 births were registered compared to an expected 183,000 births based on the crude birth rate in the 2015 population census. Hence, the government of Lao PDR is preparing to invest in improving CRVS in the country. Accordingly, this analysis employed the following data sources as main sources for producing vital statistics: the 2015 Population and Housing Census [13], the 2017 Lao Social Indicator Survey (LSIS) [14], and civil registration. Other data sources, such as the demographic surveillance system and sample registration with verbal autopsy, are nonexistent, and health facility data on the notification of births and deaths are patchy since Lao PDR’s legal framework prior to the 2018 Family Registration Law precluded health facilities from providing notification of births and deaths to the Ministry of Home Affairs, which is responsible for civil registration. The advantages and limitations of each of the selected data sources are shown in Table 1, while Table 2 shows a comprehensive list of the types of vital statistics and their corresponding data sources. There are two columns for civil registration data in Table 2: one (labeled “2018 Civil Registration”) for the types of vital statistics that can be generated based on the current system and another (labeled “Functional Civil Registration”) for what can be generated when the CRVS system is fully established in Lao PDR in the next 5–20 years.

**Table 1:** Pros and cons of data sources for vital statistics

Data source	Attributes
Population-based surveys	In countries where civil registration is not complete and accurate, population-based surveys such as the Demographic and Health Surveys or Multiple Indicator Cluster Surveys are employed. However, these surveys have limitations, such as not having the ability to provide the full range of vital statistics, having higher sampling errors since it uses a sample of the population, not being able to provide data for monitoring at lower administrative levels such as districts, and lacking yearly data since they are carried out every 3 to 5 years. Other drawbacks are cost and operational requirements to conduct these types of surveys.
Civil registration	Civil registration, when complete, provides all data for vital statistics pertaining to births, deaths, marriages, and divorces on a yearly basis; it provides disaggregated data at lower administrative levels and for subgroups of the population. Unlike population-based survey and population census, civil registration provides continuous stream of data at the lowest administrative levels and for the whole population.
Population census	Like civil registration, population census provides disaggregated data, but because the census is typically done every 10 years, projections which are not entirely accurate are used in the intercensal years, and it does not provide data on maternal deaths unless verbal autopsy is incorporated. Provides cross-sectional data for the whole population every decade.

**Table 2:** Types of vital statistics and sources of data

Vital statistics	2015 Census	2017 Lao Social Indicator Survey	2018 Civil Registration	Functional Civil Registration
Birth statistics				
Absolute number of births	Yes		Yes	Yes
Crude birth rate	Yes			Yes
Sex ratio at birth	Yes			Yes
Fertility rates (general fertility rate, adolescent birth rate, age-specific fertility rates, and total fertility rate)	Yes	Yes		Yes
Low (or very low) birth weight (percent)		Yes		Yes
Preterm live births (percent)				Yes
Death statistics				
Absolute number of deaths	Yes		Yes	Yes
Crude death rate	Yes			Yes
Age-, sex-, and cause-specific death rates	Yes			Yes
Life table and life expectancy	Yes			Yes
Infant mortality	Yes	Yes		Yes
Under-five mortality	Yes	Yes		Yes
Maternal mortality ratio	*			Yes
Marriage statistics				
Absolute number of marriages	Yes		Yes	Yes
Age-specific distributions and rates	Yes			Yes
Median age at marriage	Yes			Yes
Divorce statistics				
Absolute number of divorces	Yes		Yes	Yes
Age-specific distributions and rates	Yes			Yes
Median age at divorce	Yes			Yes
Cause of divorce	Yes			Yes

*The census produced pregnancy-related deaths and not maternal deaths

#### Identifying and measuring the outcomes of the alternatives to be evaluated

Jimenez-Soto et al. [8] suggested that for the “quantity of data,” the units of records should be used for each data source as an outcome measure when the country has plans to improve CRVS, but that if there are no plans to improve CRVS, then the number of people captured in each data source should be used. Lao PDR belongs to the former category. Thus, the number of units of records in each of the three data sources was used for this analysis. The estimated total population of 6.7 million was considered the number of units for census and civil registration, but the total respondents of 52,460 in the 2017 LSIS was used as the number of units.

Regarding the quality of data, instead of using the five quality attributes of vital statistics (accuracy, relevance, comparability, timeliness, and accessibility) that Jimenez-Soto et al. [8] adapted from Mahapatra et al. [5], this analysis employed the attributes in the definition of quality of vital statistics in the United Nations (UN) Principles and Recommendations for a Vital Statistics System [15]: *completeness (or coverage)*, *correctness or accuracy*, *availability*, and *timeliness*. In addition to these attributes, this analysis included the ability to provide disaggregated data, that is, by sex, age, nationality, ethnicity, geographical area, and urban versus rural in line with Sustainable Development Goal Target 17.18 (by 2020, enhance capacity-building support to developing countries, including for least developed countries and small island developing states, to increase significantly the availability of high-quality, timely, and reliable data disaggregated by income, gender, age, race, ethnicity, migratory status, disability, geographic location, and other characteristics relevant in national contexts). We scored these attributes on a scale of 0–1. The definitions of these attributes and scoring of the data sources for each of these attributes are shown in Table 3.

**Table 3:** Data quality scores

Quality attributes	Definition	2015 Census^a^	2017 Lao Social Indicator Survey	Functional Civil Registration
Coverage or completeness	Geographical area that the data covers/sample size	*0.5*	*0.007* ^*b*^	*0.9*
Correctness or accuracy	All data items are accurately and completely recorded with no missing items	*0.5*	*0.2* ^c^	*0.9*
Availability	Data that have been collected, filed, processed, and stored in each system (CRVS) are accessible to users in a user-friendly format upon request	*0.5*	*0.5*	*0.9*
Timeliness	Data for vital statistics is generated annually	0.1	0.2	1
Disaggregated data	Data are available by sex, age, nationality, ethnicity, geographical area, income, and urban vs rural	0.5	0.5	0.9
Total score		2.1	1.4	4.6
Composite quality index		0.42	0.28	0.92

^a^Census was rated 0.5 for most of the quality attributes because of the reduction in the quality of data during the intercensal period. The score of 0.1 for timeliness represents 1 out of 10 years

^b^The score of 0.007 represents the fraction of the population interviewed, that is, 52,460/6,700,000. It is assumed that the survey sample is representative of the population

^c^It is assumed that the survey data are accurate in the year that the data was collected, but over 10 years, it is 2/10 accurate

#### Identifying and measuring the costs of the alternatives to be evaluated

The actual costs obtained by the World Bank for the 2015 Census and 2017 LSIS were US$9.3 million and US$1.2 million, respectively. The Ministry of Home Affairs’ 5-year estimated cost for the proposed Lao PDR CRVS project for building a functional electronic CRVS system is US$20 million. However, it is anticipated that after the initial major investments, such as establishing a civil management information system and generating demand for civil registration services, the recurrent costs in the subsequent years would be lower. Thus, the estimated cost for each data source over a 20-year period (equivalent of two cycles of population censuses) was computed, and the average cost per year was used in the analysis (Table 4). It is assumed that a 20-year period would be reasonable enough for the long-term benefits of a functioning CRVS system to accrue to the government and people of Lao PDR.

**Table 4:** Costs of data sources for vital statistics over a 20-year period

	1st 5 years Cost (US$)	2nd 5 years Cost (US$)	3rd 5 years Cost (US$)	4th 5 years Cost (US$)	Total for 20 years (US$)	Average cost per year (US$)
CRVS	20,000,000	9,820,000	4,820,000	4,820,000	39,460,000	*1,973,000*
Census	9,300,000	9,300,000	9,300,000	9,300,000	37,200,000	*1,860,000*
Survey	1,200,000	1,200,000	1,200,000	1,200,000	4,800,000	*240,000*

#### Conduct an economic assessment of the alternatives to be evaluated

For the CEA, the number of units/records, the quality scores (Table 3), and the cost per unit (or capita) (Table 4) were used to compute the cost per quality-adjusted capita (Table 5).

**Table 5:** Costs of data sources for vital statistics over a 20-year period

Data source	Composite quality index	Average cost per year	Number of records/units	Cost per quality-adjusted unit	Ranking
CRVS	0.92	1,973,000	6,700,000	0.32	1
Census	0.42	1,860,000	6,700,000	0.66	2
Survey	0.28	240,000	52,460	16.34	3

### Results

Of the 20 types of vital statistics, a complete and accurate CRVS system can produce all of them, the 2015 census can produce 17 of them, and the 2017 LSIS and current civil registration system can each produce 4 of them (Table 2). Regarding the average annual cost for each of the data sources over a 20-year period, CRVS was the most expensive at US$1,973,000, followed closely by population census (US$1,860,000), while population survey was much lower (US$240,000) (Table 4). Nevertheless, based on the CEA, civil registration data ranked first, followed secondly by population census, and population survey which placed third (Table 5).

### Discussion

Leaving aside the cost-effectiveness of CRVS as a data collection method, Lao PDR could also derive other economic benefits and enormous development impact from a functioning CRVS system.

Evidence suggests that countries such as Norway and Slovenia, which have well-functioning CRVS systems, do not conduct a 10-yearly population census but rather use the electronic CRVS system to carry out more frequent register-based censuses. Slovenia is reported to save about 14 million Euros by using the register-based census, which does not require enumerators going house-to-house to collect data [16]. Thus, with an improved electronic CRVS system, Lao PDR could potentially save the costs of conducting population censuses in the future.

Civil registration systems improve efficient public service delivery leading to cost savings. For example, having children’s births registered can facilitate immunization programs, especially those that require high levels of coverage to be effective. Cost can be reduced, and immunization process can be accelerated, if immunization workers can easily locate and vaccinate children when their identities and residences are known. One study in rural Mali found that the cost of immunization without an effective birth registration system was about US$2.79 per child, while the cost with registration was US$1.47 per child [17].

An effective CRVS system contributes to improving public sector governance, thereby reducing inefficiencies and waste, and could also result in cost savings. It establishes an individual’s legal identity to help determine eligibility and reduce fraud for social safety net programs. For instance, with proper identification, pensions and other social benefits will not continue to be provided to the deceased [18]. Finally, a fully functional CRVS electronic system will most likely reduce the total costs for identification systems within the public sector [18]. One of the main limitations of this study is that it assumes countries generate vital statistics from a single data source, but in reality, countries without complete and accurate CRVS systems tend to use multiple sources of data for producing vital statistics and for assessing the completeness of civil registration data.

### Conclusion

This study has shown that in the long term, improving the CRVS system in Lao PDR is more cost-effective than relying on population surveys or population censuses for producing vital statistics. The framework for economic analysis developed by Jimenez-Soto et al. [8] was adapted for the analysis using data from Lao PDR. Although the framework is not perfect, over time, as it is applied in different settings, it may be refined further and, in the future, could result in cross-country analysis.

## Data Availability

Not applicable

## Abbreviations

CEA: Cost-effectiveness analysis
CRVS: Civil registration and vital statistics
Lao PDR: Lao People’s Democratic Republic
LSIS: Lao Social Indicator Survey
UN: United Nations
